# Influence of patient satisfaction, system usability, and working alliance on depressive symptom improvement in blended cognitive behavioral therapy (bCBT): Secondary analysis of an open trial data

**DOI:** 10.1016/j.invent.2025.100815

**Published:** 2025-02-18

**Authors:** Ece Atik, Silvan Hornstein, Elisabeth Reinking, Magnus Schückes

**Affiliations:** a Translational Psychotherapy, Georg-Elias-Mueller-Institute of Psychology, University of Goettingen; b Department of Psychology, Humboldt-Universität zu Berlin, Berlin, Germany; c Faculty of Psychology and Neuroscience, University of Maastricht, Netherlands; d Institute for SME Research and Entrepreneurship, University of Mannheim, Germany

**Keywords:** Blended cognitive behavioral therapy, Depression, Working alliance, Therapy, System usability, Patient satisfaction

## Abstract

Blended cognitive behavioral therapy (bCBT), which involves the use of a digital application to support face-to-face psychotherapy, is increasingly offered to patients with depression amid a growing body of research on its efficacy. However, there is still limited understanding of the factors that influence the efficacy of this novel treatment method. To investigate the effects of potential factors such as patient satisfaction with the received treatment, patients' self-rated working alliance with their therapist, and patients' rating of system usability of the digital application, this secondary analysis study focused on a sample of 66 university students who completed an effective 6-week bCBT program that included weekly sessions with a therapist and access to a digital mental health application. We examined whether those three potential factors predict patients' improvement in depressive symptoms in a bCBT treatment. Patient satisfaction and working alliance are known predictors of treatment success in standard psychotherapy, yet their importance in blended treatment is largely unstudied. System usability is a factor that is frequently addressed while describing digital treatment programs, yet its contribution to the success of treatments has been mostly omitted. All the variables analyzed displayed a significant positive correlation with improvement in depressive symptoms. When taken together, all the factors account for 16.6 % of the variance in the outcome. However, when three variables were added in the backward multiple linear regression with stepwise elimination, only patient satisfaction emerged as a predictor of the outcome. Although there are significant correlations between working alliance and system usability and the improvement of depression in the bCBT program, their lack of predictive power in comparison to patient satisfaction renders the results inconclusive. Future studies could explore the potential contribution of additional variables to the improvement of depressive symptoms.

## Highlights

• Patient satisfaction, usability, and working alliance are associated with depressive symptom improvement in a bCBT program.
• Pairs of positive correlations among patient satisfaction, usability, and working alliance were significant.
• When three factors are analyzed together, the full model explained 16.6 % of the variance in depressive symptom improvement.
• How different predictors interact and are interlinked with one another needs further exploration.

## 1 Introduction

Digital interventions have become increasingly popular for treating mental health disorders when combined with cognitive behavioral therapy (CBT) (Erbe et al., 2017). The combination of face-to-face interactions and digital therapeutic elements is commonly referred to as blended cognitive behavioral therapy (bCBT) (Cuijpers et al., 2017). While conventional sessions involve face-to-face collaboration between patients and therapists to achieve treatment goals (Andersson, 2016; Wright et al., 2019), the technological support provided by digital interventions, such as smartphone apps, provides additional resources for patients to engage at their convenience (Philippe et al., 2022). Additional resources in the form of digital interventions may help decrease the stigma associated with mental health disorders, increase the cost-effectiveness of treatment, improve patients' symptoms and quality of life, and offer additional therapist time to focus on the patients' issues rather than psychoeducation and homework (Ebert et al., 2015; Erbe et al., 2017; Cuijpers et al., 2008; Kooistra et al., 2019; Thase et al., 2018). Yet, the inclusion of digital interventions comes with additional requirements from patients, including the ability to use computers and the internet, a greater degree of agency and self-reflection, flexibility, and the ability to control frustration. For these reasons, some patients may find these interventions challenging (Erbe et al., 2017; Rozental et al., 2015).

There exists no single definition or way of use of blended therapy in the literature (Wentzel et al., 2016). We based our definition of blended therapy in this study on the extensive review study by Erbe et al. (2017). Based on this review, blended therapy needs necessarily to involve separate face-to-face and digital components. The organization of those components has generally been classified as either sequential or simultaneous based on how the face-to-face and digital components are introduced and scheduled. Sequential bCBT treatments involve patients working individually with a digital mental health app either before or after meeting with a psychotherapist in person, such as in aftercare or stepped-care programs (Haug et al., 2015; Klein et al., 2012). Simultaneous bCBT programs offer in-person and online components within the same and single treatment period (Erbe et al., 2017). This is frequently accomplished by assigning therapeutic homework via a digital app in between therapy sessions (Berger et al., 2018; Månsson et al., 2013). Another major classification is based on the focus of the digital programs. Erbe et al. (2017) distinguish bCBT programs with an internet focus and with a face-to-face focus. The blended treatment definition addressed in this study covers a simultaneous bCBT program with a face-to-face focus since we examine the treatment context where digital intervention is offered to complement the face-to-face component and not vice versa. The central role of the psychotherapist and in-person sessions is preserved in such programs. As long as the major role of the face-to-face work with a therapist is maintained, the face-to-face component of the blended therapy may be offered in a therapist's office or online via videoconference tools, as is also commonly and increasingly being done in standard psychotherapy as well (Berryhill et al., 2019; Uysal et al., 2022). In some of the studies addressing digital mental health interventions, face-to-face therapy sessions are guided by passive therapist guidance via text messages or phone calls (Andersson et al., 2012; Forand et al., 2019; Karyotaki et al., 2022). However, this does not fall under the definition of blended therapy in this paper since it does not contain any face-to-face element.

On a global scale, depression is one of the main causes of disability (Friedrich, 2017). In Germany, depression accounts for the greatest number of sick days, and this number is still rising (Baumeister et al., 2015; Schneider et al., 2019). The lifetime prevalence of depression is around 15 % in Germany (Jacobi et al., 2004; Busch et al., 2013). The risk of depression is particularly high among university students. University students are at significant risk of depression due to academic challenges, changing lifestyles and family relationships, and post-graduation concerns (Asif et al., 2020; Beiter et al., 2015).

Previous research has evaluated the effects of bCBT on depression, showing a reduction in depressive symptoms following its implementation (Erbe et al., 2017). Moreover, bCBT has been associated with increased treatment effectiveness, optimized clinician time efficiency, and improved patient-therapist rapport and communication (Berger et al., 2018; Erbe et al., 2017; Klein et al., 2016; Thase et al., 2018). As bCBT is a still emerging intervention within psychotherapy, further research is imperative to understand its impact on mental health disorders, particularly depression since the majority of the existing bCBT programs target patients with depression (e.g., Berger et al., 2018; Schuster et al., 2020; Ly et al., 2015; Thase et al., 2018) and depression has a prominent role in leading to disability in German population (Schneider et al., 2019). Furthermore, it is essential to have a comprehensive understanding of the factors that influence the feasibility and effectiveness of bCBT to ensure its efficacy and potential.

Previous research has identified several factors that can either promote or hinder the effectiveness of bCBT (Bielinski et al., 2022; Doukani et al., 2024; Titzler et al., 2018; Waller and Gilbody, 2009). Working alliance, a therapeutic relationship that includes the bond between the patient and therapist, as well as the agreement on therapeutic tasks and goals (Bordin, 1979), has been one of those factors. Working alliance has been demonstrated repeatedly and again to be a strong predictor of patient recovery in psychotherapy; some have even proposed that it is the primary therapeutic element of psychotherapy (Baier et al., 2020; Dambi et al., 2023; Flückiger et al., 2018; Lambert and Barley, 2001). Working alliance between therapists and patients has been identified as one of those predictive factors in improvement of depressive symptoms in bCBT programs (Askjer and Mathiasen, 2021; Doukani et al., 2024; Bergman Nordgren et al., 2013; Ly et al., 2015; Vernmark et al., 2019), also mirroring the importance of therapeutic relationship in traditional psychotherapy (Horvath & Symonds, 1991). Some of this research found that a stronger working alliance in a bCBT program reported by the therapist was a significant predictor of depressive symptom improvement, whereas the alliance rated by patients was not (Askjer and Mathiasen, 2021; Vernmark et al., 2019). Other studies only investigated working alliance from the patient's perspective and found them to be a significant predictor of symptom improvement (Bergman Nordgren et al., 2013; Doukani et al., 2024; Ly et al., 2015). However, further research is needed to corroborate these findings, as the body of research in this area remains limited (Askjer and Mathiasen, 2021). System usability of the offered digital program is another potential factor in the successful delivery and subsequent effectiveness of bCBT (Maramba et al., 2019). System usability refers to the extent to which an application allows users to complete tasks efficiently, effortlessly, and satisfactorily (Bastien, 2010). A qualitative study on the usability of digital applications for depression points out that for users to fully benefit from a product's functionality, the bCBT application must be perceived as user-friendly and enjoyable (Patel et al., 2020). Therefore, it is essential to identify any potential obstacles for patients interacting with the bCBT application during its developmental stage (Jaspers, 2009). Some of the previous studies examining the efficacy of a bCBT program for depression also collected patients' assessments of the usability of the digital component and reported that the offered system has above-average usability in the standardized usability assessment scale (Atik et al., 2023; Kooistra et al., 2016). Yet, these studies only reported mean usability scores in the System Usability Scale (SUS; Brooke, 1996) and did not address its relation to the treatment efficacy. The association of usability with symptom improvement in a bCBT program is yet to be quantitatively tested. Some studies also pointed out that system usability interacts with the working alliance in a way that facilitates a higher working alliance (Doukani et al., 2020, Doukani et al., 2024), which further highlights the importance of testing usability together with the working alliance. Moreover, patient satisfaction with the offered treatment appears to be a potential factor influencing the outcome. In some of the previous studies focusing mainly on the effectiveness of bCBT, authors also reported treatment satisfaction scores of patients (Atik et al., 2023; Kooistra et al., 2016; Romijn et al., 2021). Similar to the reporting of system usability, these studies only reported patients' average satisfaction scores in the Client Satisfaction Questionnaire (CSQ-8; Attkisson and Zwick, 1982) and did not test whether satisfaction scores are related to treatment outcomes. Addressing this question is important to fill this gap and to uncover if the observed relationship between treatment satisfaction and treatment success in traditional health care (Prakash, 2010) is also reflected in the mental health context and the novel bCBT modality.

To examine the contribution of the addressed potential factors to the success of a bCBT program, this secondary data analysis focused on data from an open trial with an efficacious bCBT intervention for depression (Atik et al., 2023) and examined the influence of patient satisfaction, working alliance, and system usability on treatment outcome. Patient satisfaction and system usability have not been directly tested in relation to depressive symptom improvement earlier; yet results of qualitative studies and high usability and patient satisfaction reporting from effective bCBT programs imply a possible relationship (Atik et al., 2023; Doukani et al., 2020; Kooistra et al., 2016; Patel et al., 2020; Romijn et al., 2021). Patient-rated working alliance yielded to be a predictor of symptom improvement in bCBT programs by several studies (Bergman Nordgren et al., 2013; Doukani et al., 2024; Ly et al., 2015), although other studies did not confirm this finding (Askjer and Mathiasen, 2021; Vernmark et al., 2019), pointing out the need for clarification through further studies. In this study, we hypothesized that higher patient satisfaction with the offered treatment, working alliance, and system usability of the digital application used in the bCBT program predict improvement in depressive symptoms, indicated by reduced PHQ-9 scores.

## 2 Methods

### 2.1 Participants & procedure

This secondary data analysis was conducted as a follow-up to the feasibility and efficacy study addressing a bCBT intervention program for depression among university students in Germany (Atik et al., 2023). Students with mild to moderate symptoms of depression (ie, Patient Health Questionnaire-9 (PHQ-9) scores between 5 and 15; Kroenke and Spitzer, 2002) were enrolled in the bCBT program. Flyers displayed on campuses and student email groups at German universities in North Rhine-Westphalia were used to recruit university students. Initially, prospective participants filled out an online survey that included the PHQ-9, demographic questions, and additional questions on the exclusion criteria. In the following step, a clinical psychologist (Master of Science) conducted an in-person interview with participants who fulfilled all inclusion criteria and none of the exclusion criteria as determined by the survey evaluation. Participants underwent a 25-min video-based interview with a clinical psychologist at this stage. The two-step inclusion procedure was utilized to thoroughly evaluate sensitive exclusion criteria (such as suicidality), as well as to cross-validate self-report responses with clinical assessments.

In addition to fulfilling the PHQ-9 inclusion criteria (scores between 5 and 15), patients had to be between the ages of 18 and 65, speak German fluently, and have a smartphone with either the iOS or Android operating system and an internet connection to be eligible for enrollment in the study. The following were the exclusion criteria applied in the study: Acute suicidality (a score of two or three on item 9 of the PHQ-9), current or past bipolar disorder (ICD-10), current or past borderline personality disorder (ICD-10), current or past psychotic disorder (ICD-10), symptoms of posttraumatic stress disorder or dissociative experiences, current or past self-harm (addressed only in the screening with a therapist), (7) current or past ICD-10 alcohol or drug dependency (apart from nicotine), and (8) psychopharmacological or psychotherapy treatment during the last year.

At baseline (week 0) and post-intervention (week 6), participants completed the self-report outcome questionnaires. Additionally, a final interview was conducted with all students who had completed the bCBT intervention program to examine the facilitators and barriers to the implementation of the bCBT program (Braun et al., 2023).

In the efficacy study, 67 patients were in the completer sample of the depression intervention group (Atik et al., 2023). One participant was excluded due to incomplete responses to the questionnaires essential for this analysis. As a result, 66 students were included in this analysis (age: M = 23.65 years; SD = 3.731, range = 18–36, 87.9 % (58/66) female and 12.1 % (8/66) male). 33 % of the students were studying social sciences and humanities, 19 % studying medicine and related fields (eg, dentistry and pharmacy), 18 % science and engineering, 16 % psychology, and 5 % had other majors. 7 % of the sample were recent graduates with no information on the department.

### 2.2 Intervention: Blended cognitive behavioral therapy

The present study examined a bCBT program that included face-to-face psychotherapy and a digital component. The face-to-face component comprised six 25-min weekly sessions of individual CBT conducted via videoconferencing with a therapist. These sessions covered psychoeducation and interactive therapeutic exercises, such as creating a personal toolbox and a behavioral activation plan, and joint reflection on the tasks. The face-to-face sessions were conducted by clinical psychologists (Master of Science). For the in-session treatment manual, refer to Atik et al. (2023).

The digital element of the treatment consisted of the elona therapy smartphone application. The smartphone app provides patients access to psychoeducational materials, exercises, digital interventions, and activities that are custom-tailored to their specific symptoms during the course of the intervention. The digital materials can be adapted by the therapist according to the patient's needs. The program integrates therapeutic content into users' daily lives and is designed to increase patients' active engagement in outpatient treatment and encourage individual work between sessions (Atik et al., 2023). During this study, patients were advised to use the app for an average of 25 min each week, which is equivalent to the duration of the sessions. The available content in the elona therapy application addressing depression is described in Table 1.

**Table 1:** Content of the *elona therapy* application for addressing students with depression.

Chapter	Content
About depression	Psychoeducational content and disorder-specific knowledge about psychotherapy, the symptoms of depression, individual symptoms, comorbid conditions, and factors that promote the development and maintenance of depression
My behavior	Relationship between activities and depression, behavior analysis, and activity building and planning. Values-based work
My thoughts	Relationship between thoughts and depression. Methods of cognitive restructuring and introduction of the concept of detached mindfulness
My emotions	Relationship between emotions and depression. Psychoeducational content to perceive emotions in a more differentiated way, to understand them as indications of needs, and support in dealing with emotions
My relationships	Depression in a social context. Acquisition of social skills and competencies
Relapse prevention for depression	Methods of general relapse prevention such as recognizing individual early warning signs and building a toolbox for difficult situations
About relaxation	Psychoeducational content on relaxation techniques, focus on physical processes
Progressive muscle relaxation (PMR)	Mechanisms of action and goals of progressive muscle relaxation according to Jacobson and related exercises
Imagination	Psychoeducation on imagination exercises and related exercises
Mindfulness and meditation	Psychoeducation on awareness and meditation techniques and related exercises

### 2.3 Outcome measures

#### 2.3.1 Beck-Depressions-Inventory-II (BDI-II)

The BDI-II is a revised version of the BDI developed by Beck et al. (1996). It was validated in German by Kühner et al. (2007). This questionnaire is one of the most widely used scales to measure depression by following DSM-IV criteria. It contains 21 four-item statements to rate on a 4-point Likert scale, ranging from 0 to 3. For example, the item sadness is required to be ranked with statements ranging from 0 = “I do not feel sad” to 3 = “I am so sad and unhappy that I can't stand it”. The internal consistency is good, and the retest reliability is acceptable (Kühner et al., 2007). BDI-II was given to participants at both pre- and post-treatment.

#### 2.3.2 Working Alliance Inventory - Short Form (WAI-SR)

The working alliance between patients and therapists was measured with a widely used inventory called WAI-SR, a 12-item self-report questionnaire (Hatcher and Gillaspy, 2006; German translation: Wilmers et al., 2008). Items include statements such as “What I am doing in therapy gives me new ways of looking at my problem.” and are rated on a 5-point Likert-type, ranging from “1 = Seldom”, to “5 = Always”. There are three main categories within this inventory: Tasks, goal and bond (Hatcher and Gillaspy, 2006). The WAI-SR indicates good internal consistency. The WAI-SR was given to participants only at post-treatment.

#### 2.3.3 System Usability Scale (SUS)

The system usability was measured with the System Usability Scale (SUS) which includes 10 items with five respond options ranging from “1 = strongly disagree” to “5 = strongly agree” (Brooke, 1996; German translation: Gao et al., 2020). Examples items are “I think that I would like to use this system frequently” and “I found the various functions in this system were well integrated”. The inventory aims to measure the usability of products and services such as hardware, mobile devices, or apps. In previous research, the SUS indicates between acceptable and good reliability. The convergent validity with other assessments of perceived usability is acceptable (Mol et al., 2020). The SUS was given to participants only at post-treatment.

#### 2.3.4 Patient Satisfaction Questionnaire (CSQ-8)

The Client Satisfaction Questionnaire (CSQ-8) measures patient satisfaction with 8 questions (Attkisson and Zwick, 1982; German translation and validation: Schmidt et al., 1989). Items are rated on a 4-point Likert scale, e.g., “How satisfied are you with the amount of help you have received?” is rated from 1=”Quite dissatisfied” to 4=”Very satisfied”. Previous studies have shown that internal consistency of the CSQ-8 is good (Schmidt et al., 1989). The CSQ-8 was given to participants only at post-treatment.

### 2.4 Data analysis

Data analysis was performed with the SPSS Version 28.0. First, to identify whether the SUS, the WAI-SR, and the CSQ-8 are associated with the change in depression scores (pre- to post-change in the BDI-II total scores), correlations between change in depression scores and the three predictor variables were calculated separately. For the predictor variables where a significant correlation between a predictor and the outcome is present, a Multiple Linear Regression (MLR) (Backward Elimination Technique) was done to identify firstly the contribution of all those predictors together and secondly to examine which predictor contributes significantly. In the Backward MLR, all predictors under examination are entered into the model at the same time. Then, the model removes the least significant variable in each case until the significant variables are left. This allows us to infer which predictors explain the outcome the best. Moreover, multicollinearity-the potential for significant correlation between several independent variables- among the predictors was checked with the variance inflation factor (VIF). In cases where gender, age, and study course are significantly correlated with the outcome variable, these variables were considered confounders and included as controlling variables.

## 3 Results

### 3.1 Descriptive statistics

Table 2 depicts the descriptive statistics of age, pre- and post-measurement of BDI-II, change in depression scores, working alliance, system usability, and patient satisfaction.

**Table 2:** Descriptive Statistics for the variables Age, Pre- and Post-measurement BDI-II, Change in depression scores, Working alliance, System usability, and Patient satisfaction.

Variable	N	M	SD	Skewness	Kurtosis
1. Age	66	23.65	3.73	1.08	0.99
2. Pre-BDI-II	66	19.33	7.5	0.28	0.03
3. Post-BDI-II	66	13.33	7.5	0.48	−0.39
4. Change in BDI-II	66	6	8.60	0.34	−0.14
5. Working Alliance	61	3.98	0.813	−0.88	0.00
6. System usability	63	74.29	15.22	−0.41	−0.35
7. Patient satisfaction	65	26.17	4.2	−0.52	−0.38

*Remarks:* M = Mean, SD = Standard deviation; Pre-BDI-II = Pre-Measurement of the BDI-II, Post-.

BDI-II = Post-Measurement of the BDI-II; Change in BDI-II = Change in depression scores pre- and post-measurement.

Working alliance (*r* = 0.32, *p* = 0.011), system usability (*r* = 0.28, *p* = 0.029), and patient satisfaction (*r* = 0.43, *p* < 0.001) all had a weak to moderate significant correlation to depressive symptom improvement as demonstrated by the change in the BDI-II total scores. Moreover, a positive correlation between the working alliance and system usability (*r* = 0.61, *p* < 0.001), working alliance and patient satisfaction (*r* = 0.76, *p* > 0.001), and system usability and patient satisfaction (*r* = 0.54, *p* = 0.001) was found (Table 3 depicts an overview of the results). As the correlation between working alliance and patient satisfaction is above 0.7, indicating a strong correlation, a collinearity diagnosis was performed to check for multicollinearity in the regression. There was no significant correlation between gender, age, the study course, and the change in depression scores, as well as no significant correlation between gender, age, the study course, and the three factors. Hence, no controlling variables needed to be included in the regression.

**Table 3:** Bivariate Correlations among the constructs, Change in depression scores, Working alliance, System usability, and Patient satisfaction.

Variable	N	M	SD	1	2	3
1. Change in BDI-II	66	6	8.60	–		
2. Working Alliance	61	3.98	0.813	0.32⁎	–	
3. System usability	63	74.29	15.22	0.28⁎	0.61⁎⁎	–
4. Patient satisfaction	65	26.17	4.2	0.43⁎⁎	0.76⁎⁎	0.54⁎⁎

*Remarks:* Change in BDI-II = Change in depression scores pre- and post-measurement.

⁎ *p* < 0.05

⁎⁎ *p* < 0001. N = sample size, M = mean, SD = standard deviation.

### 3.2 Backward multiple linear regression

In the backward multiple linear regression technique, all the variables under examination were fitted into a multiple linear regression model first, which was the first stage in backward elimination. From this first model, the variable with the highest *p*-value was removed and then a new model was fitted with the remaining variables. This approach was continued until the *p*-value for each variable in the model fell below a predefined threshold, which in our case was 0.05. This methodical approach was preferable for our purposes as it fits the three predictor variables into the same model, minimizes the number of predictors that could have an impact on the result, lessens the issue of multicollinearity, and addresses overfitting (Hocking, 1976; Neter et al., 1996). With the predictor variables in our investigation, multicollinearity is likely to arise, given that each pair derived by three predictor variables had moderate to strong significant correlations with one another (see also Table 3). MLM can address that several factors influence a single outcome (improvement in depression) in this study and that the relationships are complex given that all predictor variables are components of the same treatment program.

The MLR revealed that the Full Model Fit explains the change score significantly, *R*^*2*^ = 0.166., *F* = 3.73, *p* = 0.02. Hence, 16.6 % of the variance in the change in depression score is explained by all variables together. However, their independent contribution to the change score is not significant. Table 4 depicts the results of the individual predictors of working alliance, system usability, and patient satisfaction on the outcome. None of the scores explain the change in depression significantly. There is only a trend for patient satisfaction.

**Table 4:** Multiple Regression to predict improvement of depressive symptoms predicted from working alliance, system usability, and patient satisfaction.

Model 1				
Variable	β	*t*	*p*	*VIF*
Working alliance	0.14	0.07	0.946	2.74
System usability	0.04	0.43	0.672	1.6
Patient satisfaction	0.72	1.89	0.065	2.4

*Remarks: N* = 66. β = Beta coefficient. VIF = Variance inflation factor.

MLM (backward procedure) shows that when working alliance, system usability, and patient satisfaction were put together in the model with the aim of elimination, in other words, when they competed with each other, only patient satisfaction emerged as a significant predictor of the change in depression scores (*β* = 0.81, *p* = 0.001). The remaining model explains 16.3 % of the variance significantly, *R*^*2*^ = 0*.*16.3, *F* = 11.28, *p* = 0.001. Hence, it can be concluded that patient satisfaction with the bCBT treatment predicts the improvement of depressive symptoms. While the model was significant, patient satisfaction alone was not significant, although it approached the significance level (*p* = 0.065) (see Table 4). This suggests and highlights the importance of patient satisfaction among the variables we have examined in the model in this study in explaining the significant model, yet it leaves room for potential other variables we could not examine in this study influencing patient satisfaction and influencing the significance of the model.

## 4 Discussion

This study aimed to investigate to what extent working alliance, system usability, and patient satisfaction in a bCBT treatment predict improvement in depressive symptoms. The results indicate a collective influence of these three predictive factors, which accounted for 16.6 % of the variability observed in the improvement of depressive symptoms. Furthermore, results from correlation analyses underscored significant positive correlations between the improvement of depressive symptoms and the strength of the working alliance, patient satisfaction, and system usability of the digital app in the bCBT program. Upon closer examination through regression analysis, it was found that when all three factors were considered together, only patient satisfaction was a significant predictor. This result does not negate the potential contribution of working alliance and system usability to symptom improvement. Rather, it suggests that some of their effects may be subsumed within the broader construct of patient satisfaction. Furthermore, the strong correlation observed between patient satisfaction and working alliance suggests that some of their combined effects could potentially be intertwined in explaining variance in working alliance. In this regard, the findings are consistent with previous research, which revealed that to conceptualize a patient component in the effectiveness of a bCBT program, additional elements are required, and the effects are often intertwined (Doukani et al., 2020, Doukani et al., 2024). This research found this mainly for working alliance, implying that working alliance cannot be established without sufficient usability of the digital program. Similarly, we discovered in our research that patient alliance is a complex concept that cannot be conceptualized without other components of a bCBT program, potentially also including working alliance and usability of the digital application, but not limited to them. Also, while the model with three predictors investigated predicted 16.6 % of the variance in the outcome, the remaining model with patient satisfaction predicted 16.3 %. This underscores that patient satisfaction plays a special and relatively more prominent role than other predictors investigated in this study. Psychotherapists who practice bCBT and further investigations on the predictors of patient improvement should dedicate focused attention to better understand and facilitate patient satisfaction with the provided treatment. Thus, while the importance of patient satisfaction as a predictor is highlighted, the intricate interplay of patient satisfaction and its multifaceted elements require further exploration to unravel their nuanced contributions to the bCBT outcome.

In our study, correlations between pairs of predictors, such as the positive correlation between working alliance and patient satisfaction, working alliance and system usability, and patient satisfaction and system usability were found. The correlation between working alliance and system usability replicates earlier studies (Doukani et al., 2020, Doukani et al., 2024). The correlation between working alliance and patient satisfaction has vast evidence in the medical context (Kelley et al., 2014; Shay et al., 2012; Step et al., 2009). Yet, to our knowledge, such a relationship has not been directly tested in the psychotherapy practice, possibly because it may sound too straightforward. Our study also confirms such a hypothesis. The correlation between patient satisfaction and working alliance argues against experiences of patients and therapists in a qualitative study that usability of the digital application was unrelated to the therapeutic relationship (Bielinski et al., 2022). Yet in this qualitative study, both therapists and patients felt that the digital program was not sufficiently integrated into the treatment, which may explain their finding.

In the broader context of scant empirical research in this area, these findings have important implications. The existing body of research has yielded inconsistent results regarding the interplay between working alliance and the change in mental health outcomes. Divergent perspectives have emerged. For example, some scholars have reported a predictive relationship between the patient-rated quality of the working alliance and subsequent changes in outcomes in the context of bCBT (Bergman Nordgren et al., 2013; Doukani et al., 2024; Ly et al., 2015) a pattern supported by the findings of our study. In contrast, other research has emphasized the prominent role of therapist-rated alliance in determining bCBT outcomes, while de-emphasizing the predictive capacity of patient-rated working alliance (Askjer and Mathiasen, 2021; Vernmark et al., 2019). This underscores the critical importance of the therapist-rated alliance, which our study does not directly address by focusing solely on the patient-rated alliance. Consequently, future research should address the influence of therapist-rated working alliance in bCBT programs. The claim made by our study that working alliance plays a role in mediating the outcomes of blended treatments importantly contributed to the discussion on this factor. The role of working alliance on better psychotherapy outcomes has been widely acknowledged in standard CBT treatment (Baier et al., 2020; Flückiger et al., 2018; Lambert and Barley, 2001). The significant correlation between working alliance and symptom improvement in this study suggests that the established benefit of working alliance on patient improvement extends to the bCBT modality. The research addressing this in bCBT has been limited and some studies have even asserted that working alliance may not carry the same predictive weight in bCBT as it does in standard CBT (Kooistra et al., 2016). Yet, it is important to emphasize that our study was an uncontrolled trial and to uncover how far the digital part of the bCBT influences the relationship between working alliance and the change in depressive symptoms alone, further studies with a control group including a standard CBT treatment need to be conducted.

In previous studies, system usability has often been assessed to present a patient evaluation of the products used in studies addressing a bCBT intervention. Its inclusion has been instrumental in claiming that the usability of the provided digital intervention was good enough to be a barrier-free intervention for patients (Kemmeren et al., 2016; Kooistra et al., 2016; Patel et al., 2020). Remarkably, amidst its importance, to our knowledge, no study has directly addressed its relation to the outcome. The present study contributes to an understanding of the extent to which the change in depression scores is predicted by system usability. Nevertheless, it remains prudent to acknowledge the limited scope of this study, as it is tied to a specific digital application. Therefore, to be able to generalize this finding to digital applications in bCBT in general, further research is needed to replicate the finding by addressing the usability of different digital applications and their link to patients' symptom reduction. Moreover, future studies could address if a more user-friendly digital offering could lead to a reduction in drop-out rates or higher patient motivation to engage in therapy (Hentati et al., 2021).

As to patient satisfaction, little research has been done earlier on its effect on the improvement in depressive symptoms following a bCBT program. Previous studies with bCBT interventions mostly reported mean patient satisfaction scores as an indicator of perceived treatment acceptability (Kemmeren et al., 2016; Kooistra et al., 2016), without addressing its mediating role in outcomes. Such correlations of patient satisfaction with patient outcomes have been mostly addressed in standard clinical settings (Doyle et al., 2013; Dubina et al., 2009). The present study extends these findings to a bCBT context. Importantly, the relationship between patient satisfaction and change in depressive symptoms might not always be one-way. Both patient satisfaction and the improvement in depressive symptoms could influence each other, suggesting a reciprocal relationship (Dubina et al., 2009). Moreover, a good therapist-patient relationship can increase patient satisfaction, which underscores the need to evaluate it together with the link between working alliance and patient satisfaction (Dubina et al., 2009; Fuertes et al., 2009). Our study also does this, as we found a strong positive association between working alliance and patient satisfaction. Inferences on how those factors influence each other and the effect on the health outcome should be made cautiously, as our results only based on correlation analyses.

## 5 Limitations of the study

This study has several limitations that should be considered. First, the study only included 66 university students with mild depression symptoms based on their PHQ-9 scores as a sample. Due to the limited sample size, there is reduced potential power and limited possibility for the results to be generalized. Because the study only included university students, findings might not be representative of all people with depression. University students may differ from other populations in particular ways, such as age, cognitive abilities, or socioeconomic background. Moreover, since the sample only consisted of students with mild depressive symptoms based on PHQ-9 scores, the results might not be generalizable to people with moderate or severe depression or to a clinical patient group with an official diagnosis.

Second, this study was a secondary analysis study, which means that the data were collected for a different main goal and that the analysis may not have been specifically tailored to address the research question. Secondary analysis studies may not have the specificity required for investigating variables in the optimal way. Third, there are also limitations associated with using backward multiple linear regression with stepwise elimination as the method of analysis. This method may result in the exclusion of important variables that could interact with one another. Although the only factor that predicted the outcome was patient satisfaction in our analysis model, this approach may have missed complex relationships between different factors, such as the relationship between system usability and working alliance. Only 16.6 % of the variance in improvement in symptoms of depression could be explained by the three variables we have examined taken together. Even though the model was significant, only a small proportion of the variance is explained by this study, leaving more of the variation unexplained. It is possible that other relevant factors impacting the effectiveness of bCBT were not considered or quantified. That may also underlie the finding of this study while the model tested was significant, the most important factor emerged, patient satisfaction, alone was not significant. Potentially significant additional variables that we were unable to investigate in this study could be affecting patient satisfaction and the significance of the model. Fourth, although all three variables and the reduction in symptoms of depression were shown to be significantly correlated, correlations do not necessarily indicate a causal relationship. It is unclear whether these variables directly affect symptom improvement or if there are other underlying factors that could be affecting depression outcomes as well as satisfaction.

Fifth, working alliance was solely measured from the patient's perspective in this study. Prior research indicates that the alliance rated by the therapist may be a key factor in predicting outcomes (Askjer and Mathiasen, 2021; Vernmark et al., 2019). Future studies should include both patient and therapist perspectives on the working relationship and examine its influence on outcomes. Sixth, all measurement instruments used in this study were introduced post-treatment (week 6). Ratings of working alliance, system usability and satisfaction while concluding the program may be biased in a way that it does not represent patients' experience at a random time point in the treatment. Seventh, the measurement instruments used in this study were initially designed for face-to-face psychotherapy treatment contexts. These tools may not be equally suitable for a bCBT context. Therefore, to draw conclusions that are specific for bCBT context, there emerges the need to develop blended treatment specific questionnaires that measure working alliance, system usability, and patient satisfaction, and examine our findings with such specific instruments.

Lastly, this study evaluated a six-week bCBT program. This can provide insights into early effects, but it does not inform us about how predictive variables associate with longer term symptom change. To overcome this limitation, additional studies using complementary follow-up measurement points are required.

## 6 Conclusions

This study aimed to investigate the roles of patient satisfaction, working alliance, and system usability in patients' improvement in depressive symptoms within a six-weeks bCBT program in a university students' sample with elevated depression. When all three factors analyzed together in the multiple linear regression analysis, they explained 16.6 % of the variation in the outcome. While all three factors were positively correlated with symptom improvement when tested separately, only patient satisfaction emerged as a predictor in the analysis model. This suggests a role of patient satisfaction in symptom improvement and a relative importance of the patient satisfaction compared to other factors examined. Yet, it remains unclear why patient satisfaction alone was not significant on its own while the model was significant. The effects of working alliance and system usability as well as further factors that were not investigated in this study may be partly captured by patient satisfaction. Future studies are needed to uncover isolated contributions of factors on patient symptom improvement. The findings suggest that improving patient satisfaction in digital mental health interventions is important for patient symptom reduction in the bCBT treatment. Findings are important since the relationship of patient satisfaction to patient outcomes in the bCBT treatments has largely been overlooked in the literature earlier. Patient satisfaction is a component of the treatment that is a complex phenomenon and needs further exploration.

## Declaration of competing interest

This declaration addresses the manuscript titled “Influence of system usability, working alliance and patient satisfaction on depressive symptom improvement in blended cognitive behavioral therapy (bCBT): Secondary analysis of an open trial data”.

Author EA declares that she is employed by Elona Health, the manufacturer of the elona therapy app used in this study. ER and SH are employed part-time by Elona Health. MS is a shareholder of Elona Health.
